# Insights Into Oxidized Lipid Modification in Barley Roots as an Adaptation Mechanism to Salinity Stress

**DOI:** 10.3389/fpls.2020.00001

**Published:** 2020-02-04

**Authors:** Dingyi Yu, Berin A. Boughton, Camilla B. Hill, Ivo Feussner, Ute Roessner, Thusitha W. T. Rupasinghe

**Affiliations:** ^1^ School of BioSciences, University of Melbourne, Parkville, VIC, Australia; ^2^ St. Vincent’s Institute of Medical Research, University of Melbourne, Fitzroy, VIC, Australia; ^3^ Metabolomics Australia, Bio21 Institute, University of Melbourne, Parkville, VIC, Australia; ^4^ School of Veterinary and Life Sciences, Murdoch University, Perth, WA, Australia; ^5^ Albrecht-von-Haller-Institute for Plant Sciences, Department of Plant Biochemistry, University of Goettingen, Goettingen, Germany; ^6^ Goettingen Center for Molecular Biosciences, Department of Plant Biochemistry, University of Goettingen, Goettingen, Germany

**Keywords:** oxidized lipids, salt stress, barley roots, mass spectrometry, lipid modification, *Hordeum vulgare*, oxylipins

## Abstract

Lipidomics is an emerging technology, which aims at the global characterization and quantification of lipids within biological matrices including biofluids, cells, whole organs and tissues. The changes in individual lipid molecular species in stress treated plant species and different cultivars can indicate the functions of genes affecting lipid metabolism or lipid signaling. Mass spectrometry–based lipid profiling has been used to track the changes of lipid levels and related metabolites in response to salinity stress. We have developed a comprehensive lipidomics platform for the identification and direct qualification and/or quantification of individual lipid species, including oxidized lipids, which enables a more systematic investigation of peroxidation of individual lipid species in barley roots under salinity stress. This new lipidomics approach has improved with an advantage of analyzing the composition of acyl chains at the molecular level, which facilitates to profile precisely the 18:3-containing diacyl-glycerophosphates and allowed individual comparison of lipids across varieties. Our findings revealed a general decrease in most of the galactolipids in plastid membranes, and an increase of glycerophospholipids and acylated steryl glycosides, which indicate that plastidial and extraplastidial membranes in barley roots ubiquitously tend to form a hexagonal II (HII) phase under salinity stress. In addition, salt-tolerant and salt-sensitive cultivars showed contrasting changes in the levels of oxidized membrane lipids. These results support the hypothesis that salt-induced oxidative damage to membrane lipids can be used as an indication of salt stress tolerance in barley.

## Introduction

Plants have evolved complex strategies to cope with salinity stress, including the compartmentation and exclusion of Na^+^ and Cl^−^, biosynthesis of compatible solutes, and maintenance of membrane integrity and fluidity by lipid rearrangement (Allakhverdiev et al., 1999; Wu et al., 2005; Salama et al., 2007; Upchurch, 2008; Sarabia et al., 2018). In recent years, lipidomics has emerged as an effective tool to understand the diversity of membrane lipid compositions and unravel the roles of lipids in plant adaptation and tolerance to abiotic stresses (Welti et al., 2007; Natera et al., 2016; Tenenboim et al., 2016). Lipids and their related metabolites can now be readily detected from cultured cells or tissue extracts, identified and quantified on a large scale with high sensitivity using the latest techniques in lipidomics (Horn and Chapman, 2012; Köfeler et al., 2012; Haslam and Feussner, 2017; Yu et al., 2018). The detected changes in individual lipid species in stress-treated varieties can indicate the functions of lipids and related proteins or genes affecting lipid metabolism or lipid signaling (Welti et al., 2007). Previous studies on lipid composition alterations in response to various plant abiotic stresses, including drought, wounding, freezing, salinity, and nutrient deficiency, have uncovered diverse roles of membrane lipids and associated genes (Horn and Chapman, 2012; Vu et al., 2014; Tarazona et al., 2015; Narayanan et al., 2016; Natera et al., 2016; Sarabia et al., 2018; Tawaraya et al., 2018; Yu et al., 2018).

Cell membranes serve as semi-permeable barriers and gatekeepers that harbor proteins to regulate the influx of sodium ions and osmolytes (Upchurch, 2008). The adjustment of membrane fluidity partially relies on alterations of the unsaturation degree of polar glycerol-based membrane lipids (Murata and Los, 1997; Sui and Han, 2014). To maintain the stable structure of chloroplast or plastidic membranes in saline environments, plants alter their monogalactosyl diacylglycerol (MGDG) to digalactosyl diacylglycerol (DGDG) ratios; this alteration is associated with a membrane phase transition between bilayer phases and other less mobile non-bilayer phases (De Vries et al., 2004; Narayanan et al., 2016).

Salt stress can induce oxidation of vital molecules including glycerol-based membrane lipids (Mosblech et al., 2009) through the enhanced production of reactive oxygen species (ROS). Excessive ROS can react with cellular lipids and induce lipid peroxidation, which produces either esterified or free oxidized fatty acids (FAs) called oxylipins, which are considered biochemical markers of oxidative stress in plants (Howe and Schilmiller, 2002; De Azevedo Neto et al., 2006; Przybyla et al., 2008; Mosblech et al., 2009; Vu et al., 2012). The resultant oxylipins that are esterified in complex polar lipids localized in cell membranes can lead to a deterioration of membrane permeability or even cause cell death (Liu and Huang, 2000; Mosblech et al., 2009).

In addition to glycerol-based lipids, lipid remodeling of sphingolipids (SLs) and sterols (STs) under stress is gaining more attention (Michaelson et al., 2016). SLs are reported essential to basic cellular functions as well as having central roles in the signaling for programmed cell death (PCD) (Michaelson et al., 2016). Compositional changes of ceramides (Cer), hexosylceramides (HexCer), and conjugated STs have been observed under different abiotic stresses (Tarazona et al., 2015; Michaelson et al., 2016; Ferrer et al., 2017). However, the exact roles of these lipids in plant responses to stress are yet to be fully elucidated.

Recent studies have also drawn attention to cardiolipin (CL) accumulation as a response to osmotic stress (Pineau et al., 2013; Pan et al., 2014). CLs are a class of dimeric glycerophospholipids (GPs), and primarily localized in the inner membrane of mitochondria, where they account for around 10% of the total lipid content (Ardail et al., 1990; Schwarzländer and Fuchs, 2017). CLs not only contribute greatly to the permeability of the mitochondrial membranes, but also recruit soluble proteins to the membranes to stabilize respiratory chain supercomplexes (Mårtensson et al., 2017). Studies in various bacterial species, including *Escherichia coli*, *Bacillus subtilis*, and *Staphylococcus aureus*, provide evidence that an increase in CL content is a key factor in osmotic stress adaptation (Romantsov et al., 2008; Romantsov et al., 2009). To date, knowledge on the role of CL in eukaryotes, and in plants in particular, is scarce.

In this study, we present a high-throughput and high-sensitivity mass spectrometry–based lipidomics platform for comprehensive characterization of membrane lipid responses that also captures a range of oxidized lipids. Previously reported workflows for structural annotation and quantification of oxidized lipids in wheat seeds have been based on accurate mass and MS/MS spectra required extensive computation to process the data (Riewe et al., 2017). Our new approach is based on accurate mass combined with scheduled Multiple Reaction Monitoring (sMRM); the resulting data allow structural identification based on accurate mass of the precursor, observed product ions for oxidized lipids with retention time (RT) followed by a simple data processing workflow (Yu et al., 2018). We applied this method to profile oxidized lipids in roots of four Australian barley cultivars (two feed varieties Mundah and Keel which are salt-tolerant; and two malting varieties Gairdner and Vlamingh which are salt-sensitive) in response to salt stress (Coventry et al., 2008; Yu et al., 2018). Barley (*Hordeum vulgare* L.) is an excellent model plant for investigating salt stress, as it is the most salt-tolerant cereal (Munns and Tester, 2008) and different varieties of barley vary in their response to salinity stress (Shelden et al., 2013). Recent studies on two barley varieties (Clipper vs. Sahara) with contrasting response to salt stress have displayed a diversity of lipid alterations using lipidomics approaches (Natera et al., 2016). Spatial lipidomics based on imaging mass spectrometry (IMS) has also revealed differential lipid profiles under salt stress in barley roots and seeds (Sarabia et al., 2018; Gupta et al., 2019; Sarabia et al., 2019) The investigation of lipid profiles across different genotypes with contrasting salinity tolerance levels will lead to a better understanding of existing genetic diversity in current crop germplasm and shed more light into so-far unexplored functions of lipids in salinity tolerance. Our results deliver new insights into the impact of salt stress on the oxidized membrane lipids of barley roots and possible mechanisms of salt adaption of cereal crops.

## Material and Methods

### Growth Condition, Salt Treatments, and Biomass Measurements

Four varieties of barley were selected based on their importance for crop production, commercially relevant traits, and known difference in germination phenology and salinity tolerance (Tavakkoli et al., 2012; Kamboj et al., 2015; Sarabia et al., 2018; Yu et al., 2018). This selection comprised of two Australian barley feed (Mundah, Keel) and two malting genotypes (Gairdner, Vlamingh). Seeds of four Australian barley (*H. vulgare* L.) cultivars Mundah, Vlamingh, Keel, and Gairdner were provided by the University of Adelaide (SA, Australia). Cultivars were selected based on prior knowledge of their contrasting responses to salinity stress (Coventry et al., 2008; Cao et al., 2017; Yu et al., 2018). Barley seedlings (20 plants per variety and treatment) were grown in hydroponics for 5 weeks as previously described (Cao et al., 2017; Yu et al., 2018). Seeds were imbibed in deionized water with aeration for 16 h and then transferred to moistened filter paper for vernalization at 4°C. After 2 days, seeds were transferred to a plant growth chamber (Fitotron, Weiss Gallenkamp, UK) with 16 h light and 8 h dark for 4 days with the temperature set to constant 17°C. After germination, seedlings were transplanted into a hydroponic system as previously described (Shavrukov et al., 2012). Seedlings were distributed randomly to avoid systematic errors. The nutrient solution was a modified Hoagland’s solution with pH adjusted to 6.5 (Hoagland and Arnon, 1950) and replaced weekly to reduce microbial contamination and to avoid nutrient depletion. Salt treatment was initiated 7 days after germination (when the third leaves had just emerged) and was carried out in four 25 mM NaCl increments per day until a concentration of 250 mM NaCl. This was reached by adding 25 mM NaCl into the growth chamber every 6 h in each day over 2.5 days.

A supplement of 6.4 mM CaCl_2_ was added to the nutrient solution to maintain free Ca^2+^ levels for salt-treated plants (Tester and Davenport, 2003). Control and salt-treated plants were harvested on a single day after a 5-week growth period. Plants were divided into two groups: the first group comprised 40 plants which were used for biomass measurement (five replicates per variety and treatment); the second group consisted of 80 plants from which the roots were harvested for lipid analysis (five replicates per variety and treatment and two random plants per biological replicates).

Barley roots from both groups were quickly separated from shoots with sterilized scissors, gently washed with distilled water to remove remaining hydroponics solution, and blotted dry. Fresh weight (FW) of roots from the first group was immediately measured using an electronic balance (BW 420H, Shimadzu Corporation, Japan). Roots were placed into paper bags and oven-dried at 70°C for 48 h for dry weight (DW) measurements. Roots from the second group were immediately snap-frozen in liquid nitrogen and stored at −80°C until extraction.

### Chemicals and Lipid Standards

Methanol (LC-MS grade) was purchased from Fisher Scientific (Scoresby, VIC, Australia); Hexane (LC grade) was from Honeywell (Taren Point, NSW, Australia), and 2-propanol (LC-MS grade) was from RCI Labscan (Bangkok, Thailand). Deionized water was produced by a Millipore Milli-Q system (Billerica, MA, USA). Standards of PE(12:0/12:0) and Cer(d18:1/12:0) were purchased from Avanti Polar Lipids (Alabaster, Alabama, US). A mixture of the two lipid standards was prepared as a stock solution at a concentration of 1 mM in methanol/chloroform 1:1 (v/v) and stored at −20°C. All other chemicals were purchased from Sigma-Aldrich (Castle Hill, NSW, Australia).

### Lipid Extraction

For lipid extraction, frozen roots were homogenized into a fine powder using a mortar and pestle and liquid nitrogen. Lipids were extracted according to the procedure previously described for sphingolipid extraction in plant tissues (Markham et al., 2006). Homogenized frozen barley root powder (250−300 mg, exact weight of each sample was recorded) was quickly resuspended with a monophasic mixture of 2-propanol/hexane/water 60:26:14 (v/v/v, 6 ml) and incubated at 60°C for 30 min in an Eppendorf Thermomixer Comfort (Hamburg, Germany) mixing the solutions at 500 rpm. Samples were vortexed for 10 s and sonicated for 1 min every 10 min during incubation. The extracts were centrifuged at 2,000 g for 20 min at room temperature. The supernatant was transferred to a new tube, evaporated to dryness under a stream of nitrogen, then reconstituted in 500 μl of 2-propanol/methanol/water 4:4:1 (v/v/v) and stored at −20°C. A total of five biological replicates with each replicate combining two random plants were prepared. In order to compensate for variations in sample preparation and ionization efficiency, a total of 10 μl of an internal standard mixture, consisting of 100 μM of PE(12:0/12:0) and Cer(d18:1/12:0), was spiked into each replicate prior to extraction. A pooled biological quality control (PBQC) sample was produced by collecting 150 μl from each replicate as described previously (Hill et al., 2014). PBQC samples were used to monitor the reproducibility within and between different sample batches.

### HPLC-ESI-QqTOF Conditions

Chromatographic separation of all lipid species was carried out using an Agilent Poroshell column EC-C18 (100 × 2.1 mm, 2.7 μm) at a flow rate of 0.40 ml/min at 50°C. Three linear gradients based on two mobile phases—mobile phase A, methanol/20 mM ammonium acetate 3:7 (v/v); and mobile phase B, 2-propanol/methanol/20 mM ammonium acetate 6:3:1 (v/v/v)—were applied for different lipid classes as published earlier (Yu et al., 2018). The eluted lipids were detected using a SCIEX TripleTOF™ 6600 QqTOF mass spectrometer (Framingham, Massachusetts, USA). The 6600 TripleTOF™ was equipped with a Turbo V™ dual-ion source (ESI and APCI) and an automated calibrant delivery system (CDS). Three HPLC-ESI-QqTOF based PRM assays established as described were applied to profile all lipid species however, in addition oxidized lipids were analyzed based on precursor-product ion transitions (Yu et al., 2018).

The 6600 TripleTOF™ was calibrated automatically every 10 samples *via* the CDS delivering APCI calibration solution (Foster City, CA, USA). CDS injected either positive or negative APCI calibration solution depending on the polarity of ESI and was used to calibrate the mass accuracy of the 6600 TripleTOF™ system in both ionization modes including TOF-MS (MS1 scan) and high-sensitivity MS/MS. With calibration, the mass resolution for precursor ions in MS1 spectra was ∼35,000 FWHM, while the resolution for the resulting fragments in high sensitivity MS/MS scans (PRM transitions) was ∼20,000. Actual mass accuracy was below 5 ppm in MS1 spectra and 10 ppm in MS/MS spectra.

Five biological replicates per treatment per variety were prepared and analyzed in the same LC-MS batch. As a result, 423 of 517 targeted lipids were detected with CV values of PBQC samples below 25% and used for further analysis. Most of the remaining 84 compounds with CVs above 25% were of low abundance and were not included in further analysis. Median CV values of the 423 compounds as an indicator for analytical reproducibility was 9%, which is well within acceptable limits for lipidomics. Detailed 423 lipid species profiles for all treatments and varieties have been included in **Supplementary Data File S1**.

### Data Processing

#### Mass Feature Extraction From MS1 Data

MarkerView software (Version 1.2, SCIEX, Framingham, Massachusetts, USA) was used to extract mass features from both positive and negative ion mode MS1 data. Mass features were extracted for ions with an *m/z* range between 100 and 1,600 and eluting between 0.5 and 16 min. Noise threshold was set at 300. RT and *m/z* alignment of the mass features were performed with tolerances of 5% and 0.01 Da, respectively. Intensities were normalized by a manual scale factor, which was calculated from an internal standard intensity and sample weight. Only features that were detected in at least three samples of each group were extracted. Only features which contained an isotopic partner were selected for further data analysis. RTs were aligned using internal standards.

#### Peak Picking for Lipid Profiling Based on MS/MS Data

Lipid profiling using MS/MS data in PRM assays was based on the total peak area of extracted ion count chromatogram (EICC) for one or multiple fragment ions in MultiQuant (Version 3.0.2). For glycerol-based monoacyl (GLP) and diacyl lipids (GP), such as phosphatidylcholine (PC), phosphatidylethanolamine (PE), phosphatidylglycerol (PG), phosphatidylinositol (PI), and phosphatidylserine (PS), as well as CLs performed in negative ion mode, peak area of all negatively charged FA fragments were summed as published earlier (Yu et al., 2018); while for diacylglycerols (DAGs) detected in positive ion mode, the total peak area of all fragments resulting from neutral loss of a FA chain was used. For SPs, the sum of peak area of positively charged long chain bases (LCBs) and their dehydrates from up to three dehydration processes were used for profiling HexCer and Cer species. For STs, the dehydrated ST backbone was the only fragment chosen. Peak picking for fragment ions was finally set to 100 ppm width. Integration settings were as follows: noise percentage, 40%; Gaussian smooth width, 2 points. Peak areas were normalized based on the intensity of internal standards and sample weight.

#### Statistical Analysis

Tukey’s multiple comparison test was employed for FW and DW comparisons across all four cultivars using Prism Version 7.00 (GraphPad Software Inc., San Diego, CA, USA).

For both targeted and untargeted lipid analysis, intensities of compounds/features in each sample (control and salt-treated) were acquired and normalized to the value equivalent to 250 mg fresh sample weight. Student’s *t*-tests were conducted on each compound/feature to test for significance (*p*-value) between control and salt-treated samples in each variety using GraphPad Prism 7.00. Adjusted *p*-values were obtained with Benjamini–Hochberg false discovery rate (FDR) correction using MetaboAnalyst (Version 3.0). Panel bar plots and heat maps based on lipid data were created using the R software environment 3.5.0 (http://cran.r-project.org/).

## Results

### Impact of Salinity on Root Biomass Accumulation

**Figure 1:**
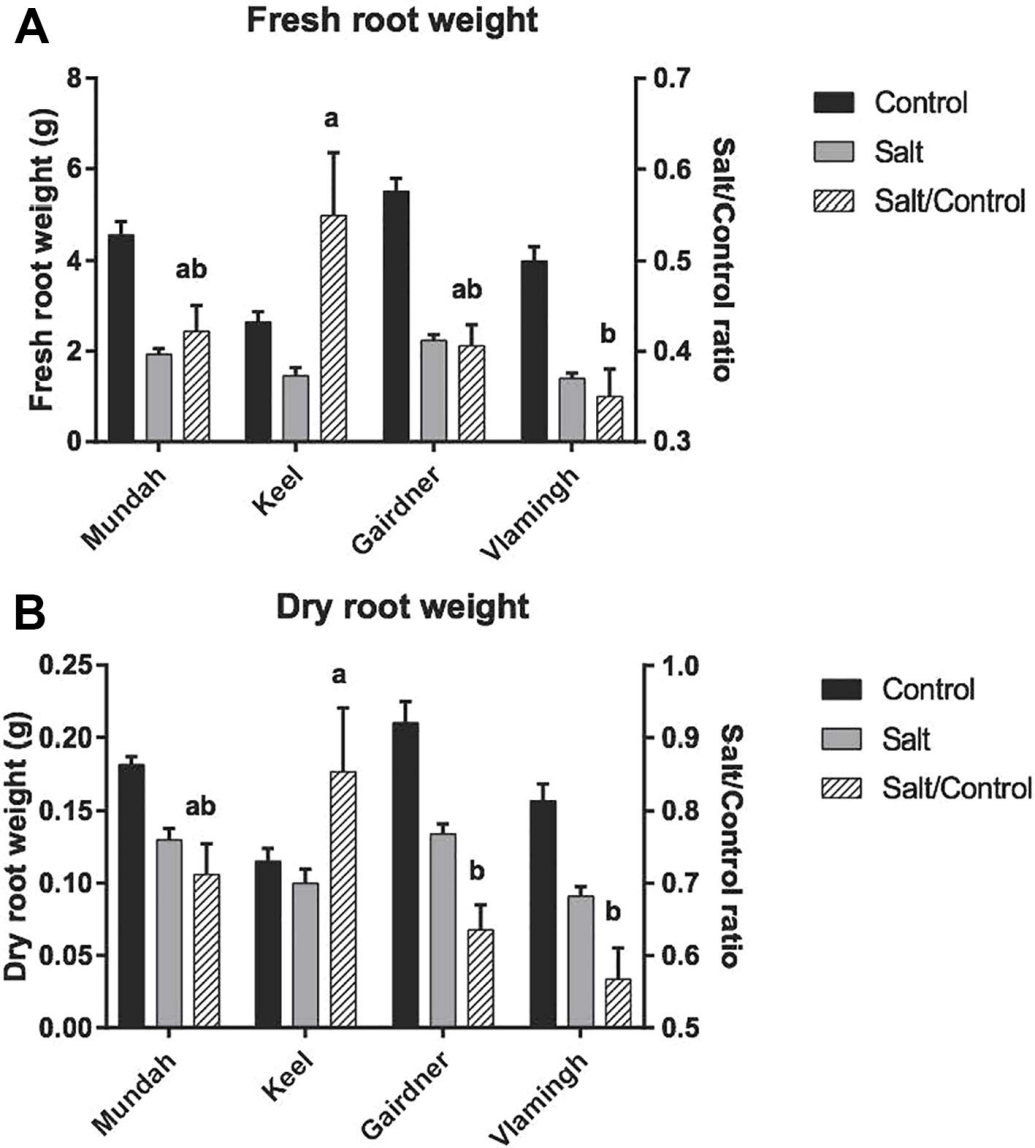
Fresh root weight **(A)** and dry root weight **(B)** of control and salt-treated barley roots across four varieties. Letters above the error bars denote significant differences between salt/control ratio of varieties (Tukey’s multiple comparison test; p < 0.05, n = 4; mean ± SD). Mundah and Keel are salt-tolerant barley cultivars; Vlamingh is salt-sensitive; the salt tolerance ability of Vlamingh is between Mundah and Gairdner.

Root FW and DW decreased significantly for all cultivars in response to 5 weeks of salinity stress (**Figure 1**). Under control conditions, Gairdner had the highest root FW and DW, followed by Mundah, Vlamingh, and Keel, which had much lower root FW and DW compared to the other cultivars. As roots are the primary site of salt contact, the ability of roots to maintain FW/DW was shown to be an important indicator of the degree of salinity tolerance (Cao et al., 2017). A comparison of the ratios of values of salt to control (salt/control ratio) was used to evaluate the maintenance of biomass between different varieties. After the 250 mM salt treatment, Keel maintained the highest FW, and also had the highest salt/control FW ratio of above 0.5. Vlamingh showed a significant lower salt/control FW ratio than Keel (*p* < 0.05). The ability of Mundah and Gairdner to maintain FW was between that of Keel and Vlamingh. Salt-tolerant varieties showed a distinctly higher ability to maintain DW than salt-sensitive varieties. Keel was shown to be the most tolerant variety in maintaining root DW followed by Mundah. Vlamingh and Gairdner as salt-sensitive varieties have significantly lower root salt/control DW ratios.

### Profiling of Oxidized Diacyl-Membrane Lipids From Extracted MS1 Features

To monitor the dynamics of oxidized lipids, we extended the lipidomics workflow established previously (Yu et al., 2018) to include the measurement of oxidized diacyl-lipids. Overall, 38 oxylipin-containing diacyl-GPs (PC, PE, PG, PI and PS) and -GLs (MGDG, DGDG) were detected and profiled using the extended workflow. Due to low abundance of some lipid classes present in the samples, oxidized lyso lipids and triacyl GLs were not detected.

To identify oxidized diacyl-lipids, we first generated a theoretical list of potential oxidized lipids containing five types of 18-carbon oxidized FAs and then manually verified the presence in our samples according to precursor *m/z* match, RT pattern, and MS/MS spectra. The five types of oxylipins including 18:2-O (C_18_H_31_O_3_), 18:3-O (C_18_H_29_O_3_), 18:4-O (C_18_H_27_O_3_), 18:2-2O (C_18_H_31_O_4_), and 18:3-2O (C_18_H_29_O_4_) were selected based on their occurrence in previous studies (Ibrahim et al., 2011; Vu et al., 2012; Riewe et al., 2017). Oxidized lipids in the list either carry one of the three most abundant normal-FAs (16:0, 18:2, 18:3) with one oxylipin, or carry both oxidized fatty acyl chains. The total number of oxygen atoms in acyl chains of listed lipids range from one to four. Notably, each type of oxylipin might refer to one or several structures, which cannot be accurately identified by the mass spectrometry approach used in this study. As previously summarized, 18:4-O can identify as: oxo-phytodienoic acid (OPDA) 18:3-O, and can refer to two possible lipid structures: a keto FA and a hydroxy FA; 18:2-O and 18:2-2O are either hydroxy or dihydroxy FA acids, respectively; and 18:3-2O is possibly derived from ketols, FA hydroperoxides, or dihydroxy FAs or their mixtures (Mosblech et al., 2009; Vu et al., 2012).

**Figure 2:**
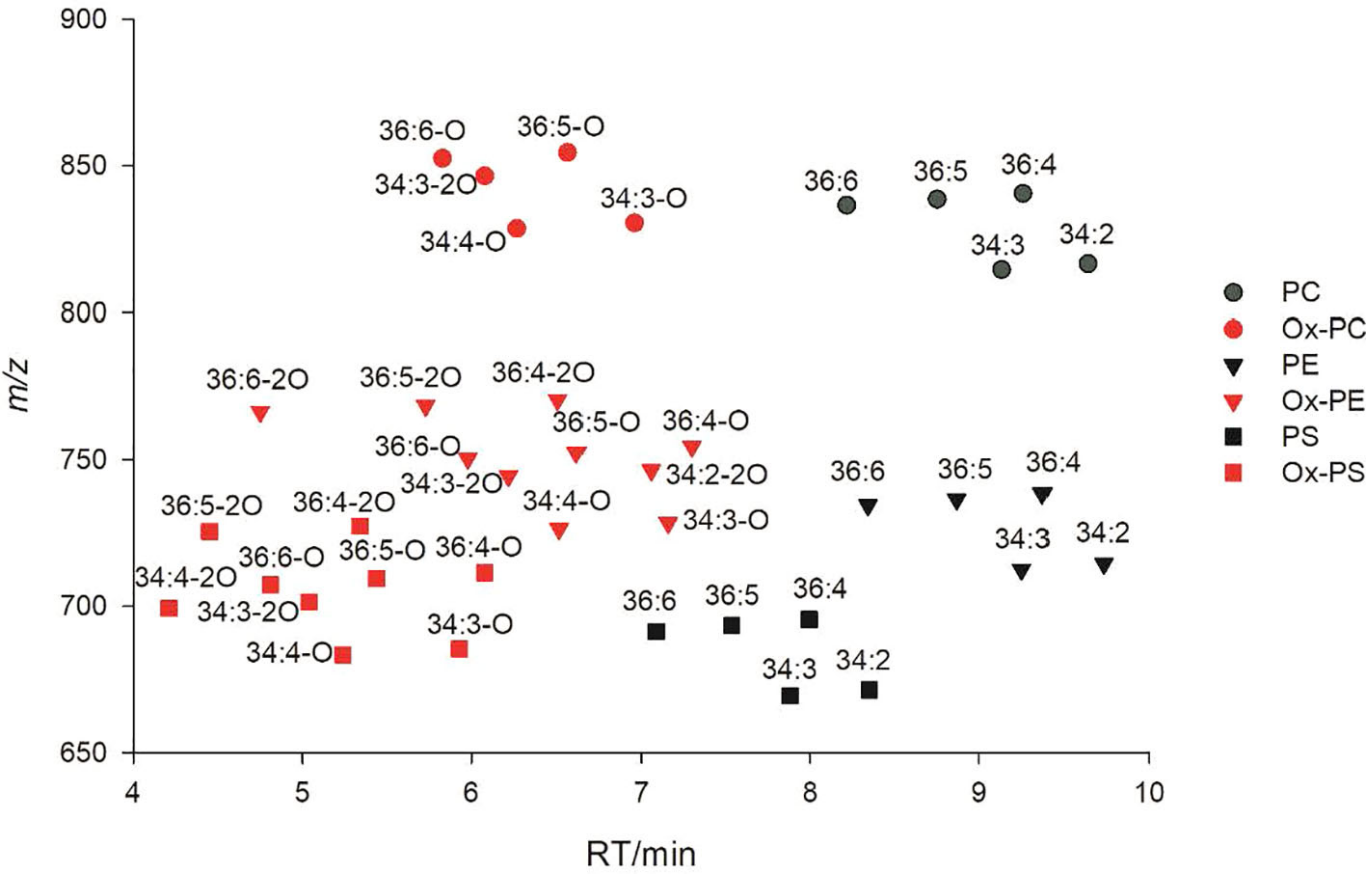
2D LC-MS plot of oxidized phosphatidylcholine (PC)/phosphatidylethanolamine (PE)/phosphatidylserine (PS) and their parent non-oxidized lipids.

Using a mobile phase containing ammonium acetate, most ox-GPs and ox-GLs will be observed as negatively charged [M+OAc]^−^ or [M−H]^−^ anions or both, which is analogous to normal diacyl species. Collision-induced dissociation (CID) of negatively charged precursors in MS/MS experiments induced cleavage of two acyl chains into carboxylate anions. The *m/z* values of carboxylate anions for different oxylipin chains are listed in **Table 1**. Specifically, fragments of oxidized FA18:3-2O and 18:2-2O were detected as 18:4-O and 18:3-O anions, respectively, due to a dehydration process. Apart from the precursor ions and fatty acyl fragments, some other characteristic fragments related to the polar head of lipids can also be observed as important indicators. Lipid separation on reversed-phase columns is largely dependent on FA composition. Elution patterns of lipid species within a lipid class is another important criterion to remove false IDs. Shorter acyl chains and higher unsaturation degree result in an earlier elution time for similar lipids (Patton et al., 1982). Oxidized lipids elute earlier than their parent non-oxidized lipids due to presence of more hydrophilic oxidized acyl chains, and RTs of oxidized lipids differing in carbon length and number of double bonds display similar pattern as their parent non-oxidized lipids (**Figure 2**).

**Table 1:** Detected oxidized diacyl glycerolipids and glycerophospholipids using HPLC-ESI-QqTOF. Detected/theoretical precursor ions, acyl fragments detected from MS/MS spectra, RTs, and mass deviations are listed.

No.	Class	Species	Detected Precursor	Theoretical Precursor	Ox-FA Fragment	FA Fragment	RT	Mass Error on Precursor
1	ox-PC	PC(34:3)-O	830.555	830.557	293.212	255.232	6.96	1.81
2	ox-PC	PC(34:4)-O	828.539	828.533	291.196	255.232	6.27	−7.12
3a	ox-PC	PC(36:5)-O	854.555	854.551	293.212	279.232	6.57	−4.21
3b	ox-PC	PC(36:5)-O	854.555	854.551	295.227	277.216	6.57	−4.21
4	ox-PC	PC(36:6)-O	852.539	852.536	293.216	277.216	5.83	−3.40
5	ox-PE	PE(34:2)-2O	746.497	746.490	293.216	255.232	7.06	−8.98
6	ox-PE	PE(34:3)-2O	744.482	744.486	291.196	255.232	6.22	4.97
7	ox-PE	PE(34:3)-O	728.487	728.483	293.212	255.232	7.16	−5.49
8	ox-PE	PE(34:4)-O	726.471	726.474	291.196	255.232	6.52	4.13
9	ox-PE	PE(36:4)-2O	770.497	770.500	293.212	279.232	6.51	3.50
10	ox-PE	PE(36:4)-O	754.502	754.500	295.227	279.232	7.3	−3.31
11a	ox-PE	PE(36:5)-2O	768.482	768.485	291.196	279.232	5.73	3.64
11b	ox-PE	PE(36:5)-2O	768.482	768.485	293.216	277.216	5.73	3.64
12a	ox-PE	PE(36:5)-O	752.487	752.488	293.212	279.232	6.62	1.59
12b	ox-PE	PE(36:5)-O	752.487	752.488	295.227	277.216	6.62	1.59
13	ox-PE	PE(36:6)-2O	766.466	766.466	291.196	277.216	4.75	0.00
14	ox-PE	PE(36:6)-O	750.471	750.472	293.212	277.216	5.98	1.20
15	ox-PG	PG(34:2)-O	761.496	761.499	295.227	255.232	6.37	3.81
16	ox-PG	PG(34:3)-O	759.481	759.474	293.212	255.232	5.64	−9.35
17	ox-PG	PG(34:4)-O	757.465	757.471	291.196	255.232	5.09	8.05
18	ox-PI	PI(34:3)-O	847.497	847.492	293.212	255.232	5.34	−6.49
19	ox-PI	PI(34:4)-O	845.482	845.479	295.227	255.232	4.76	−2.84
20	ox-PI	PI(36:4)-O	873.513	873.515	295.227	279.232	5.59	2.29
21a	ox-PI	PI(36:5)-O	871.487	871.487	293.212	279.232	4.96	0.01
21b	ox-PI	PI(36:5)-O	871.487	871.487	295.227	277.216	4.96	0.01
22	ox-PI	PI(36:6)-O	869.481	869.479	293.212	277.215	4.26	−1.96
23	ox-PS	PS(34:3)-2O	701.439	701.440	291.196	255.232	5.04	1.71
24	ox-PS	PS(34:3)-O	685.444	685.438	293.212	255.232	5.93	−8.75
25	ox-PS	PS(34:4)-O	683.429	683.424	291.196	255.232	5.24	−7.48
26	ox-PS	PS(34:4)-2O	699.424	699.421	291.196	255.232	4.21	−3.32
27	ox-PS	PS(36:4)-2O	727.455	727.452	293.216	279.232	5.34	−4.81
28	ox-PS	PS(36:4)-O	711.460	711.463	295.227	279.232	6.08	4.50
29a	ox-PS	PS(36:5)-2O	725.439	725.435	291.196	279.232	4.45	−5.24
29b	ox-PS	PS(36:5)-2O	725.439	725.435	293.216	277.216	4.45	−5.24
30a	ox-PS	PS(36:5)-O	709.444	709.440	293.212	279.232	5.44	−6.06
30b	ox-PS	PS(36:5)-O	709.444	709.440	295.227	277.216	5.44	−6.06
31	ox-PS	PS(36:6)-O	707.429	707.423	293.212	277.216	4.81	−8.91
32	ox-MGDG	MGDG(34:4)-O	825.536	825.528	291.196	255.232	7.29	−9.21
33a	ox-MGDG	MGDG(36:5)-O	851.552	851.558	293.212	279.232	7.21	7.52
33b	ox-MGDG	MGDG(36:5)-O	851.552	851.558	295.227	277.216	7.21	7.52
34	ox-MGDG	MGDG(36:6)-O	849.536	849.535	293.212	277.216	6.47	−0.82
35	ox-DGDG	DGDG(34:3)-O	989.605	989.607	293.212	277.216	6.82	2.02
36	ox-DGDG	DGDG(36:4)-O	1015.620	1015.624	295.227	279.232	7.06	3.64
37a	ox-DGDG	DGDG(36:5)-O	1013.605	1013.608	293.212	279.232	6.32	3.16
37b	ox-DGDG	DGDG(36:5)-O	1013.605	1013.608	295.227	277.216	6.32	3.16
38	ox-DGDG	DGDG(36:6)-O	1011.589	1011.591	293.212	277.216	5.54	2.17

RT, retention time; FA, fatty acid; PC, phosphatidylcholine.

**Figure 3:**
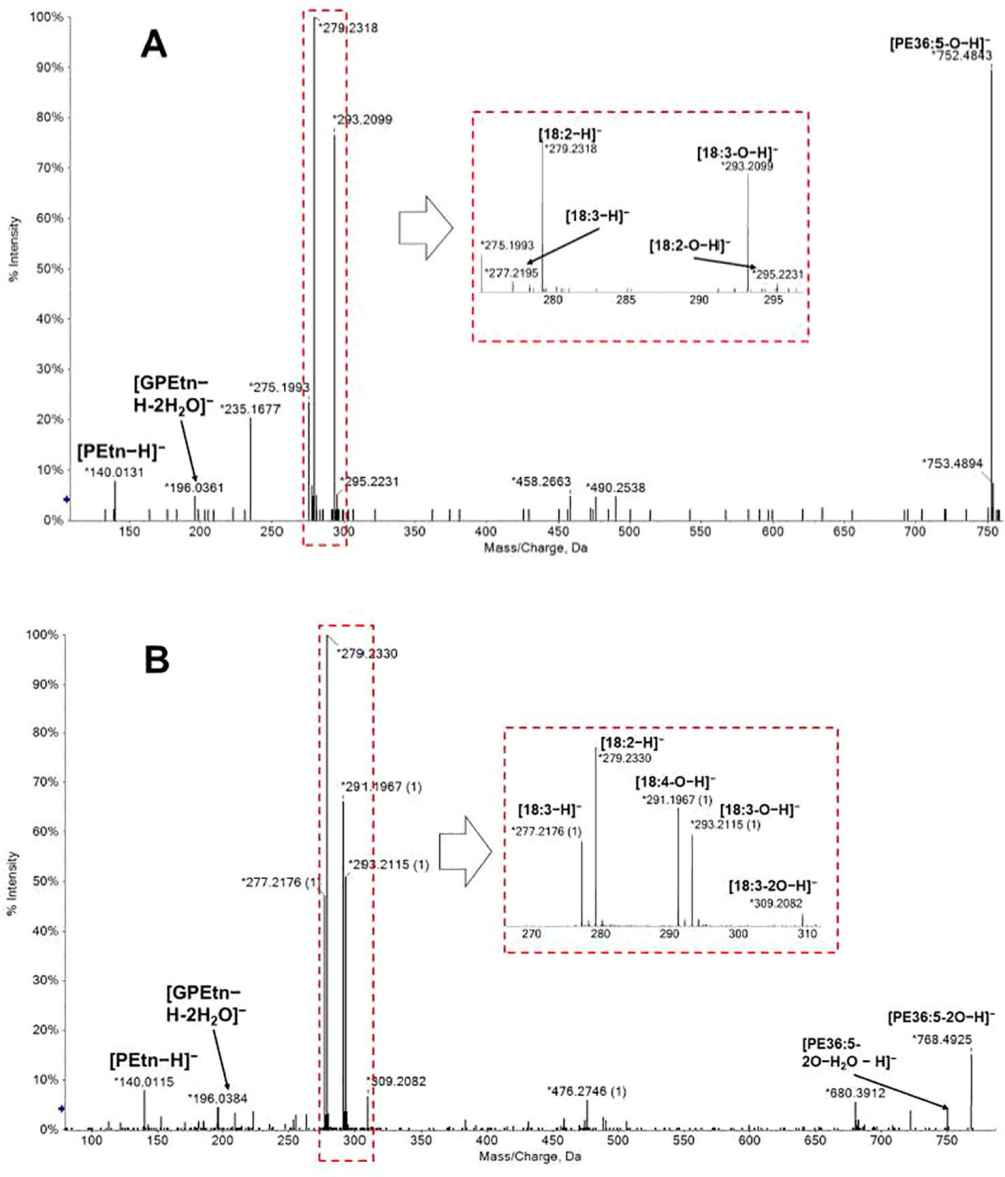
MS/MS spectra of [M−H]− precursors of PE(36:5-O) **(A)** and PE(36:5-2O) **(B)**. Acyl chains can be partially resolved in negative ion mode MS/MS spectra. PE(36:5-O) were a mixture of PE(18:2-O/18:3) and PE(18:3-O/18:2); PE(36:5-2O) were a mixture of PE(18:2-2O/18:3) and PE(18:3-2O/18:2). GPEtn: glycerophosphorylethanolamine; PEtn, phosphorylethanolamine.

Following this workflow, 38 ox-lipids were confirmed in this study, including 4 ox-PC, 10 ox-PE, 3 ox-PG, 5 ox-PI, 9 ox-PS, 3 ox-MGDG, and 4 ox-DGDG species. Molecular formula, acyl-chain fragments, RTs, and mass deviations of precursor ions in MS1 spectra of these lipids are listed in **Table 1**. Each detected ox-GP or GL molecular species consists of a normal-FA chain, 16:0, 18:3, or 18:2, in combination with an oxidized chain from 18:3-O, 18:3-2O, 18:2-O, and 18:2-2O. Representative MS/MS spectra of PE(36:5-O) and PE(36:5-2O) are shown in **Figures 3A, B**, respectively.

### Lipid Composition of Barley Roots Is Significantly Altered After Salinity Stress in All Four Varieties

The focus of this study was to investigate salt-induced alterations of membrane lipids, including GPs, GLs, ox-GPs, ox-GLs, SPs, and STs. We also included lipid species which are usually not present in membrane bilayers but potentially provide a source of backbones and acyl chains in membrane lipid synthesis, or act as signaling intermediates associated with membrane activities, such as DAGs, lyso (monoacyl)-GPs/GLs (Okazaki and Saito, 2014; Tarazona et al., 2015). The most abundant lipid profile of barley roots has been reported.

**Figure 4:**
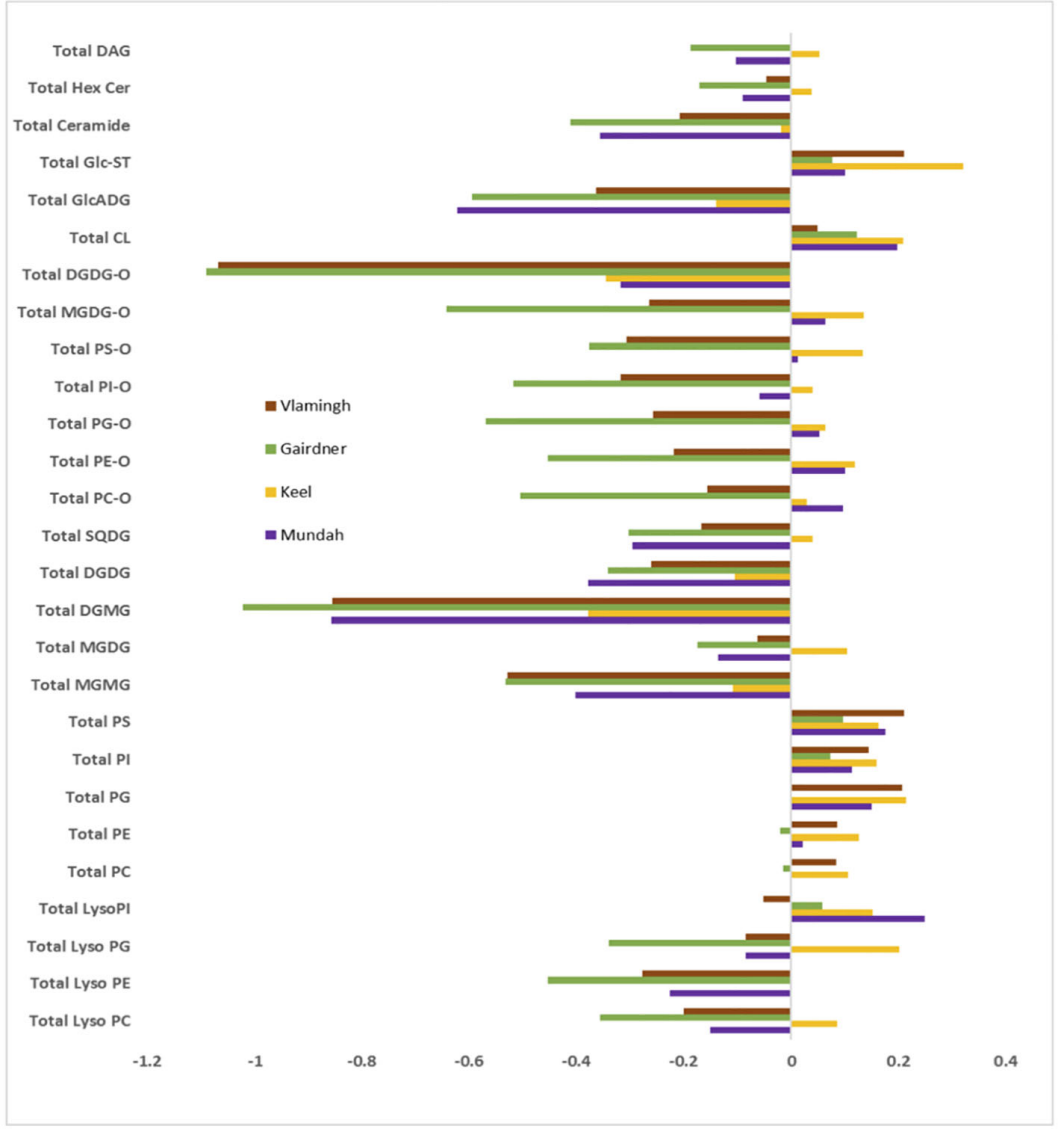
Log2 fold change (FC) of total lipids per each class after salt treatment in four barley varieties: Gairdner, Vlamingh, Mundah, and Keel.

To provide a global visualization of the lipidome alterations, the lipid data were presented as log fold change (FC) of total lipid content after salinity stress in each lipid classes for all four cultivars (**Figure 4**). FC of the total lipid content per each lipid class was calculated using the log ratio of the lipid response in salt-treated to control barley roots. The log FC of the total lipid profile for each class shows distinct profiles between control and salt-treated samples for all four cultivars, which exemplifies that the barley root lipidome is significantly altered by salt treatment (**Figure 4**). Upon salt treatment, the root lipidome of the four varieties become more similar to each other than under control conditions for several lipid classes, such as GPs, GLs, lyso-GPs, and SLs, while oxidized lipids show a significant difference between the salt stress sensitive and tolerant varieties. The FC of the total lipid content of PC, PE, PG, PI, PS, CL, and Glc-ST show a significant increase, while lysophosphatidylcholine (LPC), lysophosphatidylethanolamine (LPE), lysophosphatidylglycerol (LPG), MGDG, monogalactosyl monoacylglycerol (MGMG), DGDG, digalactosyl monoacylglycerol (DGMG), sulfoquinovosyl diacylglycerol (SQDG), glucuronosyl diacylglycerol (GlcADG), and Cer show significant decreases following salt treatment. The most significant lipid profile changes based on the varieties which are tolerant to salt stress compared to those being sensitive to salt tolerance were observed in the oxidized lipids as shown in **Figure 4**. The total oxidized lipid profile showed little increase or no change for the two salt-tolerant cultivars Mundah and Keel, whereas the salt-sensitive cultivars Gairdner and Vlamingh show significant decreases in response to salt stress. Overall, the total lipid profile of all the lipid classes clearly showed that both treatment and genotype contribute to the diversity of the barley root lipidome.

### Linolenic Acid Levels Increase in the Majority of Structural Diacyl GPs Containing Medium to Long Fatty Acyl Chains (C14~18) in Response to Salinity Stress

To further investigate how treatment modified individual lipid species levels and if there are distinct differences in the lipid levels based on genotype, pairwise Student’s *t*-test and FC analysis of GPs were carried out on the 423 GP species (including 34 oxdized lipids) between control and salt-treated samples (**Figure S1**).

GPs are considered as major structural components of plant plasma membranes and intracellular membranes. Among them, PC and PE are the two predominant structural GPs; the other GPs are mainly PG, PI, and PS species (Furt et al., 2011). One distinct feature for structural GPs observed in salt-treated roots of all four barley varieties was the increased amount of linolenic acid (18:3) across several numbers of GP species. This is reflected by the substantial accumulation of diacyl-GPs containing a linolenic acid (18:3) and a medium to long chain FA (14:0, 16:0, 16:1, 18:0, 18:1, 18:2, and 18:3), resulting in combined FA chain lengths as 32, 34, and 36 with a high number of double bonds, as they account for more than 90% of total diacyl-GPs (**Figure S1**). Our previous work provided insight of the lipid abundance in barley roots and most lipids belongs to diacyl-GPs and GIPCs in the salt-treated group were present with higher abundance than control, while more lyso-species and diacyl-GLs (except DAG) were observed to be present in higher amounts in control sample (Yu et al., 2018). Different varieties show different magnitudes in the increase of these lipids. Based on the results of the pairwise *t*-tests between control and salt-treated samples in each variety, 27, 23, and 25 out of 31 linolenic acid–containing diacyl-GPs were significantly increased (adjusted p < 0.05) in Keel, Mundah, and Vlamingh respectively; while only 9 species were significantly increased in Gairdner.

Contrasting trends were observed in other structural diacyl-GPs containing a C18:3 combined with a very long chain FA (VLCFA, C20~26) across different varieties (**Figure S1**). Different patterns were observed for the 22 lipids monitored across the four varieties. Gairdner showed a substantial decrease in most of these lipids with 20 lipids decreased and 15 that were statistically significant. Vlamingh also held an overall decrease (18 lipids) but to a lesser extent than Gairdner and only 4 being statistically significant. In the two salt-tolerant varieties Mundah and Keel, almost equal numbers of lipids decreased (12 in Mundah and 10 in Keel) and increased (10 in Mundah and 12 in Keel). In Mundah and Keel, only five and two lipids showed statistically significant decreases; three and four lipids showed statistically significant increases, respectively.

DAGs, which are critical precursors for the synthesis of PCs and PEs, did not always show concurrent trends as their corresponding PC and PEs. DAGs containing a linolenic acid (18:3), such as DAG(18:2_18:3), DAG(18:3_18:2), and DAG(16:0_18:3), increased in Keel and Vlamingh but decreased in the other two varieties.

Regarding the fate of a class of potential signaling lipids—lyso-GPs, a general decrease was observed in most of LysoPCs and LysoPEs in all four varieties with statistically significance based on Student’s ***t***-test and one-way analysis of variance (ANOVA). FDR (Hochberg and Benjamini, 1990) was used to reduce type I errors in multiple comparisons. However, several major unsaturated species are an exception with contrasting alterations across the different cultivars. For example, LysoPC(18:2) and LysoPC(18:3) in salt-treated samples showed significant increases with more than 1.40-fold in Keel, while a slight increase for LysoPC(18:2) and LysoPC(18:3) with 1.03-fold and 1.20-fold, respectively, in Mundah compared to the control samples. Conversely, a significant decrease of LysoPC(18:2) and a slight decrease of LysoPC(18:3) was detected in the two salt-sensitive varieties, Gairdner (reduced to 72% and 95% in salt-treated samples respectively), and Vlamingh (reduced to 70% and 90% in salt-treated samples respectively) in response to salinity stress.

### Decrease of Plastidial Glycerolipids in Response to Salinity Stress

GL including MGDGs/MGMGs, DGDGs/DGMGs, SQDGs, and GlcADGs are synthesized and mainly present in plastidial membranes (Dörmann, 2013; Okazaki et al., 2013). Compositional changes of these plastidial glycerolipids occur in response to drought, cold, freezing, and salinity, all of which can induce osmotic stress (Moellering et al., 2010; Tarazona et al., 2015).

In our experiments, the majority of the 111 detected GL species decreased in Gairdner (103 of the 111 species decreased of which 93 were *p* < 0.05), Mundah (103 of the 111 species decreased of which 88 were statistically significant), and Vlamingh (93 of the 111 species decreased of which 68 were statistically significant) under salinity compared to control; while a much smaller number of GL species (68 out of 111 detected of which only 34 were statistically significant) decreased in salt-treated Keel roots (**Figure S2**). On the other hand, Keel has the most species (14 species out of the 111 detected) exhibiting significant increases; while in Gairdner, Mundah, and Vlamingh only 2, 4 and 8 out of 111 detected significantly increased, respectively.

**Figure 5:**
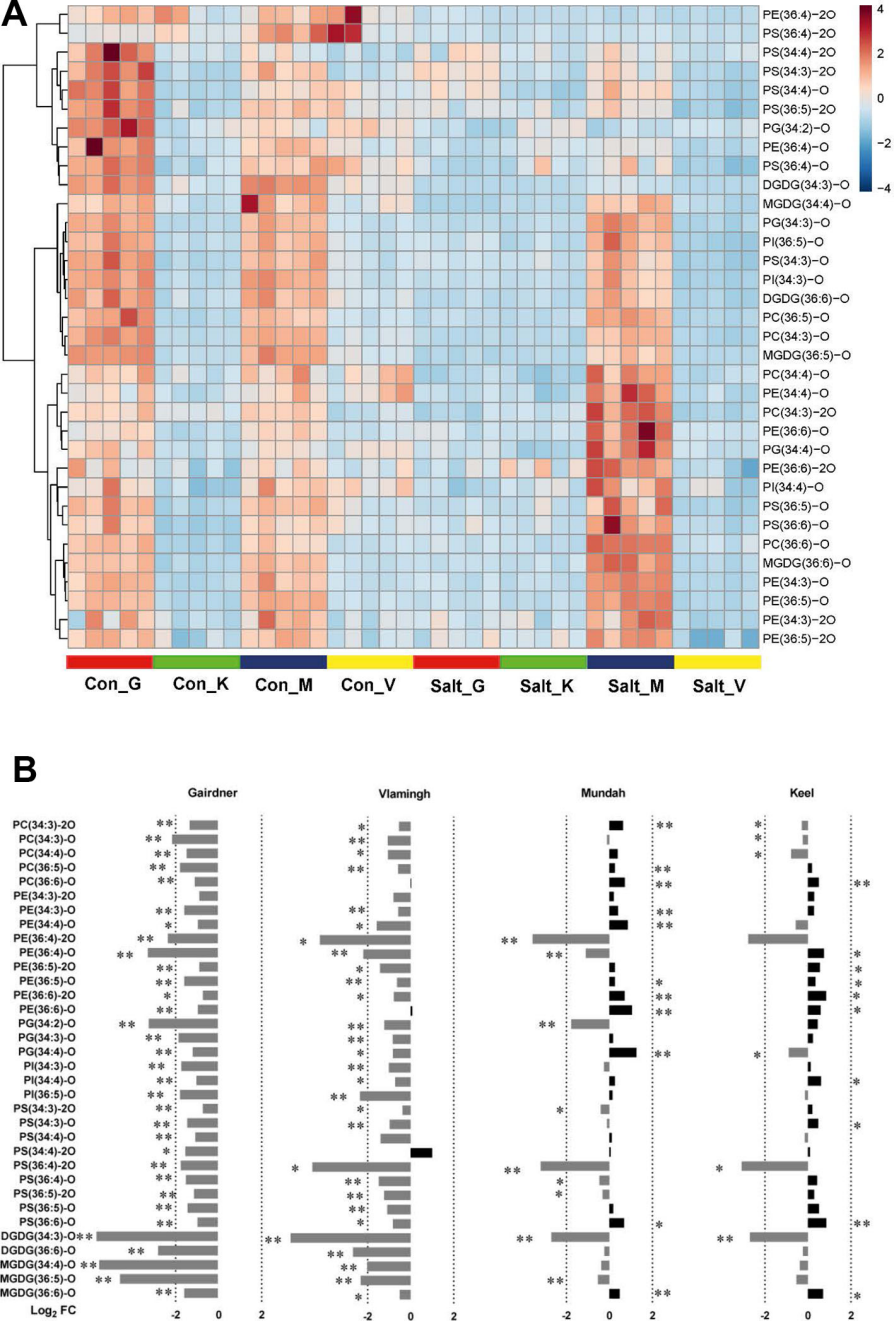
**(A)** Heat map of 34 oxidized lipids in control (con) and salt-treated (salt) samples (n = 5) in four barley varieties: Gairdner (G), Keel (K), Mundah (M), and Vlamingh (V). **(B)** Histogram displaying log2 FC of 34 oxidized lipids after salt treatment in the four barley varieties.

Compared to DGDGs, MGDGs showed a lesser degree of reduction in relative concentration across the four varieties (**Figure 5A**). For example, MGDG(16:0_18:2) reduced to 58%, 56%, and 68% in Gairdner, Mundah, and Vlamingh in salt-treated samples compared to control samples, respectively, and remained unchanged in Keel; while the level of DGDG(16:0_18:2) decreased to 28%, 20%, 19%, and 58% in Gairdner, Mundah, Vlamingh, and Keel, respectively (**Figures S2A, S2B**). The relatively smaller degree of decrease in MGDGs compared to DGDGs leads to an increase in the calculated value of the MGDG/DGDG ratio after salt stress in barley roots.

Among the four diacyl-GL classes (MGDG, DGDG, SQDG, and GlcADG), DGDGs showed the most pronounced overall decrease (**Figure S2B**). Twenty-nine out of 30 analyzed DGDG species in each of Gairdner, Mundah, and Vlamingh, as well as 24 DGDG species in Keel, decreased of which 25 species were statistically significantly decreased in Gairdner and Mundah, 20 in Vlamingh, and only 14 in Keel when compared to their respective controls. These results indicate that Keel appears to sustain the levels of DGDGs after salt treatment. However, the most abundant DGDG species, such as DGDG(16:0_18:2), DGDG(16:0_18:3), and DGDG(18:2_18:3), were still significantly decreased across all four varieties.

SQDGs and GlcADGs were observed with similar patterns in response to salinity as MGDGs and DGDGs (**Figure S2C**). Most SQDGs and GlcADGs exhibited a drastic decrease in Gairdner, Mundah, and Vlamingh; while Keel maintained levels as in control samples. Unlike structural diacyl-GPs, no obvious distinction was observed among GLs with different unsaturation degrees. Also, no obvious correlation was observed between the length of fatty acyl chains and GL modification. For example, MGDGs and DGDGs containing VLCFA did not show substantial reduction compared to those containing shorter FAs (≤ C18) (**Figure S2C**).

Monoacyl GLs including MGMG and DGMG were overall more susceptible to salt stress than diaycl-GLs. Nearly all detected DGMG and MGMG species were significantly decreased after salt treatment across all varieties, except DGMG(26:1) (**Figure S2**). In Gairdner, Mundah, and Vlamingh, all 10 species in salt-treated samples decreased to less than 30% compared to control samples; two species, DGMG(16:0) and DGMG(20:1), even dropped to below 10% in salt-treated samples. Keel again stood out with much smaller reductions of DGMG ranging between 45% and 81%.

### Salinity-Induced Accumulation of the Mitochondrial Specific Lipid Class CL

Strong accumulation was observed for the unique mitochondrial lipid class of CL in this study. In barley roots, the five most abundant CLs are CL(72:8), CL(72:9), CL(72:10), CL(72:11), and CL(72:12) (Zhou et al., 2016). After salt treatment, all five CLs showed substantial increases in Gairdner, Keel, and Mundah, where most increases being statistically significant (**Figure S3**). Specifically, CL(72:10) significantly increased 1.37-fold in Gairdner, 1.60-fold in Keel, and 1.51-fold in Mundah, while CL(72:11) significantly increased 1.76-fold in Gairdner, 1.67-fold in Keel, and 1.84-fold in Mundah (**Figure S3**). CL(72:8) increased significantly only in Gairdner (1.36-fold) and Mundah (1.63-fold). Interestingly, CL(72:9) had a significant increase only in Keel (1.78-fold). CL(72:10), CL(72:11), and CL(72:12) were also observed to be increased in Vlamingh but only CL(72:11) was statistically significant (1.35-fold) (**Figure S3**). Plant CLs are synthesized by CARDIOLIPIN SYNTHASE (CLS) transferring a phosphatidyl group from cytidine diphosphate–diacylglycerol (CDP-DAG) to a PG molecule (Nowicki et al., 2005). Based on the results from previously published studies (Zhou et al., 2016), fatty acyl chains of the five major CLs are predominantly 18:2 and 18:3. Thus, these CLs are most likely synthesized from three PGs: PG(18:2_18:2), PG(18:2_18:3), and PG(18:3_18:3). Levels of the three PGs were also significantly elevated under salt stress in Keel (1.75-fold, 1.74-fold, and 1.89-fold, respectively), Mundah (1.34-fold, 1.45-fold, and 1.61-fold, respectively), and Vlamingh (1.58-fold, 1.57-fold, and 1.57-fold, respectively) (**Figure S3**).

### Contrasting Responses of Oxidized Lipids in Response to Salinity Between Tolerance and Sensitive Barley Cultivars

Heat maps were utilized to visualize the compositional differences of oxidized lipids between treatments and across different varieties (**Figure 5A**). One distinct feature of oxidized lipids is that large variations exist among the four varieties even before and after salinity treatment. In non-treated barley roots, oxidized lipids were at higher levels in roots of Gairdner and Mundah relative to those of Keel and Vlamingh. When the varieties were exposed to salt stress, contrasting alterations to the oxidized lipid ratios were observed across the different cultivars (**Figure 5B**). In the salt-sensitive cultivar Gairdner, all of the oxidized lipids exhibited dramatic decreases after salt stress; 33 out of 34 detected species decreased significantly (adjusted *p* < 0.05); among them, 29 species decreased by more than half. In the other salt-sensitive variety Vlamingh, most of the oxidized lipids were also decreased compared to control samples but to a lesser extent than Gairdner with only 29 species out of 34 being statistically significant (**Figure 5B**). However, a general tendency of increased responses was observed in oxidized lipids in the two salt-tolerant varieties, especially in the oxidized diacyl-GP species. Twenty out of 29 detected oxidized diacyl-GPs in Mundah showed a relative increase; 10 of the 20 lipids increased with statistical significance. In Keel, 19 of 29 oxidized diacyl-GPs were also increased but to a lesser extent, with 9 of the 20 lipids exhibiting statistical significance (**Figure 5**).

## Discussion

### Root Biomass Is a Good Indicator of Salt Tolerance in Barley

High salinity (250 mM) caused significant inhibition to root growth and significantly decreased fresh and DW accumulation in all four barley varieties. The degree of decrease in both FW and DW was smaller in the salt-tolerant varieties Mundah and Keel compared to the salt-sensitive varieties Gairdner and Vlamingh. This is in agreement with previous investigations where Mundah and Keel were found to have greater salt tolerance than Gairdner and Vlamingh, as determined by maintaining their growth (Coventry et al., 2008; Cao et al., 2017).

Although both Mundah and Keel showed a strong ability to maintain root biomass, they are very likely to utilize different salt tolerant mechanisms. Mundah has been demonstrated to maintain higher Na^+^ and K^+^ concentration in roots compared to Keel (Shelden et al., 2013), which suggests that Mundah may have a greater tissue tolerance to sodium ions, while Keel may primarily rely on other mechanisms such as sodium exclusion. It has been reported that the ionic stress stage is much less evident in Mundah than in Keel, which is most likely owing to the higher ionic tolerance in Mundah (Harris et al., 2010). The distinct membrane lipid composition observed between Mundah and Keel both before and after treatment provides support for this hypothesis (**Figure 5A**). As the lipid layers in root plasma and intracellular membranes play critical roles in regulating flux of Na^+^ and K^+^ ions across membranes, the different lipid compositions may relate to different activities in the cross-membrane ion transport (Parida and Das, 2005).

### Both Unsaturation Degree and Carbon Length Distribution of GPs Are Important in Plasma Membranes Adapting to Salinity Stress

Diacyl-GPs, which are core components of plasma membrane bilayers, have been the major focus in previous abiotic stress studies (Los and Murata, 2004). The degree of unsaturation of these lipids was considered as a decisive factor in maintaining plasma membrane fluidity during stress (Upchurch, 2008). Higher concentrations of unsaturated fatty acyl chains can lead to stronger steric hindrance between diacyl-GPs, and therefore generate increased molecular disorder within a membrane bilayer (Los and Murata, 2004). Our results in the present study show that all four barley varieties increased the levels of linolenic acid (18:3)–containing diacyl-GP species, thereby increasing membrane fluidity in response to salinity. These results are in accordance with previous studies that show that the ability to maintain or increase unsaturated lipids correlates with a high level of salinity tolerance (Lin and Wu, 1996; López-Pérez et al., 2009; Mansour et al., 2015). The increase of linolenic acid in plasma membranes was also reported under salt stress in roots of other barley varieties (Yu et al., 1999; Natera et al., 2016). Therefore, we can regard it as a common salt-responsive strategy adopted in barley.

One advantage of the lipidomics method used in this study is that the composition of acyl chains at the molecular level was determined. This enabled us to precisely profile the 18:3-containing diacyl-GPs and allowed individual comparison of lipids across varieties. By taking advantage of this, we found that the increase of 18:3 was biased in favor of diacyl-GP species containing medium to long chain FAs (C14 ~ 18); while when 18:3 was found in combination with VLCFAs, contrasting alterations were observed. This suggests that there are possibly different roles of VLCFAs and medium to long chain FAs in their ability to modify the membrane biophysical properties in response to salinity. The difference may result from the higher steric hindrance and higher hydrophobicity generated by VLCFAs compared to medium to long FAs. A higher concentration of VLCFAs can lead to lower membrane fluidity (Bach and Faure, 2010). Another possible reason for the preference for a combination of 18:3 with medium to long FA is the more complex biosynthetic pathway of VLCFA which requires more biosynthetic steps, more enzymes, and energy, thus being energetically more costly for the plant (Millar et al., 2000).

### Increase of MGDG/DGDG Ratio and ASG Concentrations Suggests That Salinity May Induce the Formation of a Hexagonal II Phase in Plastid and Plasma Membranes

Compared to plasma membranes, plastid membranes seem to be less well-maintained. All four barley varieties showed a remarkable reduction in MGDG/MGMG, DGDG/DGMG, SQDG, and GlcADG amounts in response to salinity stress. This suggests a possible inhibition or even degradation of plastid membranes upon salinity. The decrease of these plastidial lipids and inhibition of galactosyl transferases combined with impairment of plastid/chloroplast membranes has been reported for salt stress before (Müller and Santarius, 1978; Shu et al., 2013; Bejaoui et al., 2016). The MGDG/DGDG ratio is associated with the physical state of plastid envelope membranes, and a higher MGDG/DGDG ratio suggests that a proportion of liquid-crystalline membranes are transformed into a hexagonal II (HII) non-bilayer phase (Dörmann and Benning, 2002; Dörmann, 2013). Lipids in the HII non-bilayer phase are organized into inverted micelles with the polar head groups on the inside and the hydrophobic tails on the outside (Jouhet, 2013). We observed a similar increase in the ratios and interpreted that this may be a possible protective mechanism of plastid membranes to maintain function under salinity stress. As previously reported, activities of plastidial enzymes such as violaxanthin de-epoxidase (VDE) require a lipid inverted hexagonal structure to operate efficiently in chloroplast membranes (Jouhet, 2013) and the VDE was also demonstrated to be expressed in wheat roots under salt stress (Zhang et al., 2003; Remorini et al., 2009).

SGs and ASGs are two classes of ST conjugates located mainly in the plasma membrane. STs and their conjugates were proposed to play a prominent role in regulating membrane acyl chain ordering and permeability (Schuler et al., 1991). Similar to MGDGs in the plastid membrane, ASGs and SGs can also induce the plasma membrane and the tonoplast to form an HII phase under freezing stress and cold acclimation (Webb et al., 1995). In the present study, The combination of the increased ASG content, MGDG/DGDG ratio, and the increased amount of ASG indicate that plastidial and extra-plastidial membranes in barley roots are shifted towards favorable conditions to form an hexagonal II phase to maintain membrane functions under salinity stress. Compared to ASGs, SGs showed fewer changes in all four barley varieties. Previously, an increase of SGs was observed in heat and freeze induced osmotic stress in *Arabidopsis* and wheat (Tarazona et al., 2015; Narayanan et al., 2016; Tamura et al., 2016). It is suggested that ASG species are much more effective in promoting the phase transition than SGs and free STs (Grille et al., 2010). Thus, the bias of favoring increasing ASG rather than increasing SG in response to salt stress might be an indicator of salt tolerance in plants. However, the specific roles of ASGs and SGs in salinity response remain to be further elucidated.

### Accumulation of CL Might Be Associated With an Altered Morphology of Mitochondria During Salt Stress

CLs, which are enriched in the inner membranes of mitochondria, were highly salinity-responsive in all barley varieties showing a remarkable increase in the present study. It appears that salinity induces the synthesis of CLs in barley roots, as their synthetic precursors, the PGs, were also observed to significantly increase.

Mitochondria are the main site of respiration, and they play crucial roles in a plant’s response to abiotic stresses, such as providing energy and reductants for stress resistance, regulating the production and removal of ROS, and serving as a source of metabolic intermediates (Millar et al., 2011). In high saline environments, the ultrastructure of mitochondria has been observed to have an enlarged and swollen appearance in the roots of *Zea mays* (maize) (Nir et al., 1970). Further experiments on *Saccharomyces cerevisiae* (baker’s yeast) demonstrated that the ability of yeast to resist the swelling under osmotic stress greatly depends on CLs found in the mitochondrial membrane (Koshkin and Greenberg, 2002). Thus, the increased CL synthesis in the present study is most likely associated with altered morphology of mitochondria during salt stress. However, further evidence for potential modifications of the ultrastructure of barley root mitochondria under salt stress is needed to demonstrate whether levels of CLs are correlated with the ability to resist the mitochondria swelling in barley roots.

Another possible mechanism underlying the increased CL synthesis may be due to induced PCD under salinity induced hyperosmotic stress as shown in yeast (Koshkin and Greenberg, 2002). Enhanced interactions between CLs and certain mitochondrial proteins can promote mitochondrial scission and breakdown of mitochondrial outer membranes, thus promoting the process of PCD (Gonzalvez and Gottlieb, 2007).

### Increased Oxidized Glycerolipids May Relate to Higher Oxidation Tolerance in Salt-Tolerant Varieties

Salinity stress can trigger ROS production in plastids, apoplasts, cytoplasm, and mitochondria and the ability to both regulate ROS production and detoxification is crucial in salt-tolerant varieties (Miller et al., 2010). Oxidized membrane lipids are an indicator of oxidation that may have occurred on membranes (Berger et al., 2001; Przybyla et al., 2008). In the present study, the levels of most of the detected oxidized diacyl-GPs showed significant increases in the salt-tolerant varieties Keel and Mundah under salt stress. MS/MS spectra of these oxidized diacyl-GPs showed that oxidized fatty acyl chains were derived from the two most abundant unsaturated FAs 18:2 and 18:3. Thus, the induced synthesis of 18:3 may not only contribute to the increase of 18:3-containing diacyl-GPs in plasma membranes to adjust fluidity, but it also may act as substrates to scavenge ROS levels and prevent oxidization of other cell components in salt-tolerant varieties. Contrastingly, the salt-sensitive varieties Vlamingh and Gairdner exhibited decreases in the concentrations of most of the oxidized diacyl-GPs. This is supported by recent studies that demonstrated that certain degrees of oxidation of membrane lipids can prevent or retard oxidative impairment to other components of the cell (Vu et al., 2012). Further, unsaturated FAs and especially FA 18:3 in membrane lipids can serve as a sink for ROS (Vu et al., 2012; Mène-Saffrané et al., 2009). They can immediately scavenge ROS through non-enzymatic reactions without activating gene or signaling pathways thus providing a fast mechanism for ROS scavenging (Mène-Saffrané et al., 2009). Thus, the alteration of oxidized lipids could be a potential indicator that differentiate salt-tolerant and salt-sensitive barley varieties.

## Conclusions

High salinity (250 mM NaCl) leads to significant alterations in the profiles of GPs, GLs, SLs, and ST derivatives in roots of four hydroponically grown barley varieties (Keel, Mundah, Gairdner, and Vlamingh). All four varieties responded to high salinity stress by remodeling of lipids in plasma membranes and intracellular membranes. In plasma membranes, the increased proportions of 18:3-containing diacyl-GPs were substantial; further, short to medium chain FAs (C14~18) had distinct responses as compared to VLCFAs. In plastid membranes, a general decrease in most of MGDGs/MGMGs, DGDGs/DGMGs, SQDGs, and GlcADGs was observed. The combination of the increased MGDG/DGDG ratio and an increased amount of ASG suggest that plastidial and extraplastidial membranes in barley roots ubiquitously tend to form an HII phase under salinity stress. Accumulation of CLs occurred in mitochondrial membranes, suggesting for a potential role of CLs in maintaining the morphology of mitochondria under salinity. In addition, salt-tolerant and salt-sensitive cultivars showed contrasting trends in the levels of most of the detected oxidized membrane lipids. Further studies need to investigate whether the behavior of these oxidized lipids can present an indication of salt tolerance levels in barley. These findings could be further investigated by future experiments to discover the insight into the biological system. In summary, our studies demonstrated new evidence on diverse alteration of lipids of cell membranes in barley roots under salinity stress, which provides promising avenues for future research to decipher the exact roles of these lipids under salt stress.

## Data Availability Statement

All datasets generated for this study are included in the article/**Supplementary Material**.

## Author Contributions

TR, BB, CH, and UR designed and planned the experiments. DY ran the experiment and collected data. TR, BB, CH, DY, UR, and IF performed data interpretation and wrote the manuscript.

## Conflict of Interest

The authors declare that the research was conducted in the absence of any commercial or financial relationships that could be construed as a potential conflict of interest.

## Abbreviations

PBQC, pooled biological quality control; ASG, acylated sterol glucoside; CDS, calibrant delivery system; CE, collision energy; Cer: ceramide; CL, cardiolipin; DAG, diacylglycerol; DGDG, digalactosyl diacylglycerol; DGMG, digalactosyl monoacylglycerol; GIPC, glycosyl inositol phosphorylceramide; GL, glycerolipid; GlcADG, glucuronosyl diacylglycerol; GlcCer, glycosylceramide; GluCer, glucosylceramide; GP, glycerolphospholipid; HexCer, monohexosyl ceramide; IPC, inositol phosphoryl ceramide; MGDG, monogalactosyl diacylglycerol; MGMG, monogalactosyl monoacylglycerol; PC, phosphatidylcholine; PE, phosphatidylethanolamine; PG, phosphatidylglycerol; PI, phosphatidylinositol; PS, phosphatidylserine; SG, sterol glycoside; SL, sphingolipid; SQDG, sulfoquinovosyl diacylglycerol; SQMG, sulfoquinovosyl monoacylglycerol.
