# Supplementary material for: Insights Into Oxidized Lipid Modification in Barley Roots as an Adaptation Mechanism to Salinity Stress
Source: Front Plant Sci. 2020 Feb 4;11:1. doi: 10.3389/fpls.2020.00001 (PMC7011103; doi:10.3389/fpls.2020.00001)
Supplement: Supplementary file 1 [file DataSheet_1.pdf]

Supplementary Material

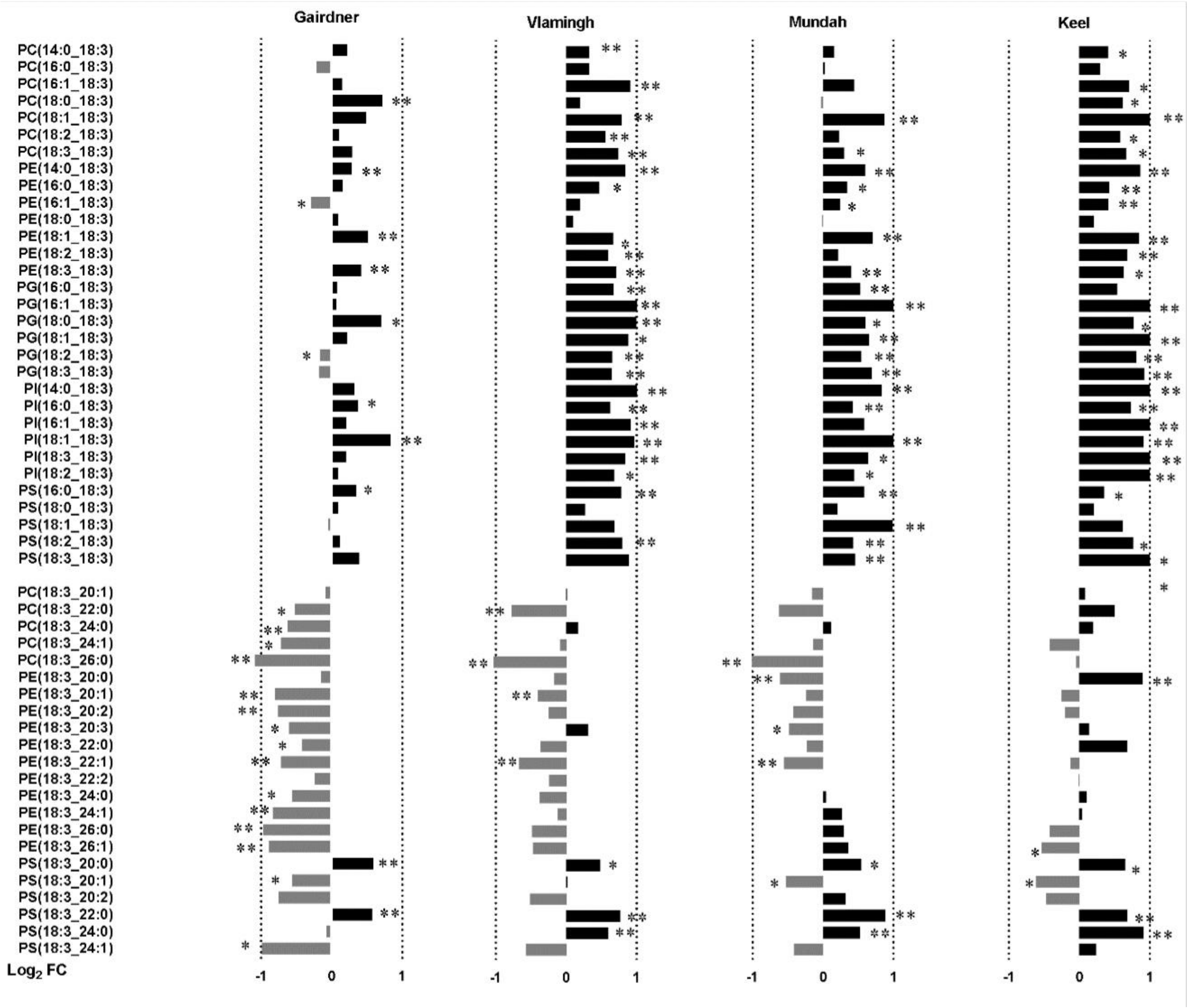

Supplementary Figure 1. Log2 fold change (FC) of linolenic acid (18:3)-containing diacyl glycerophos- pholipids (GPs) after salt treatment in four barley varieties: Gairdner, Vlamingh, Mundah and Keel. A general increase was observed in diacyl-GP species containing 18:3 and a medium to long fatty acid (C14~18, shown in upper half of the figure); while a general decrease when containing 18:3 and a long chain fatty acid (>C20, shown in lower half of the figure). Values

with significant differences between control and salt-treated samples are la- belled with asterisks (*: adjusted $p < 0.05$; **: adjusted $p < 0.01$). The ±2-fold change is indi- cated by dashed lines.

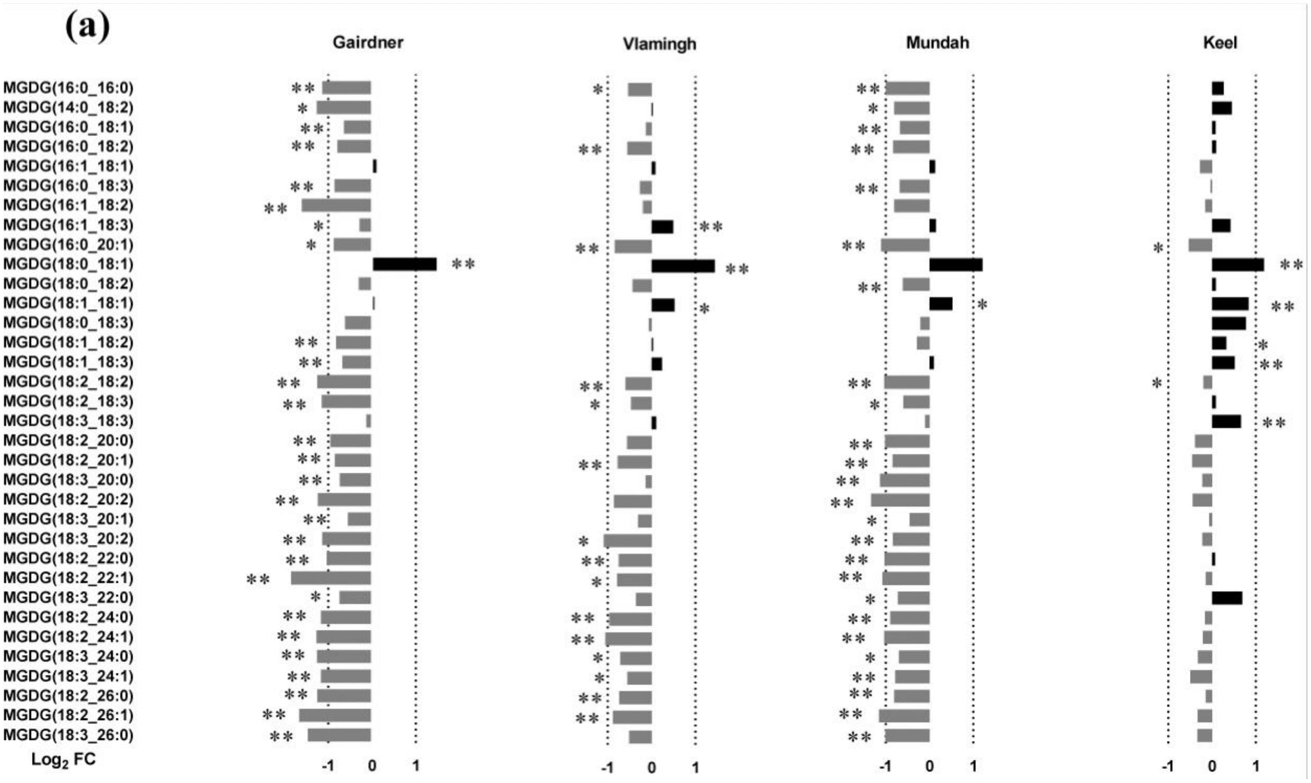

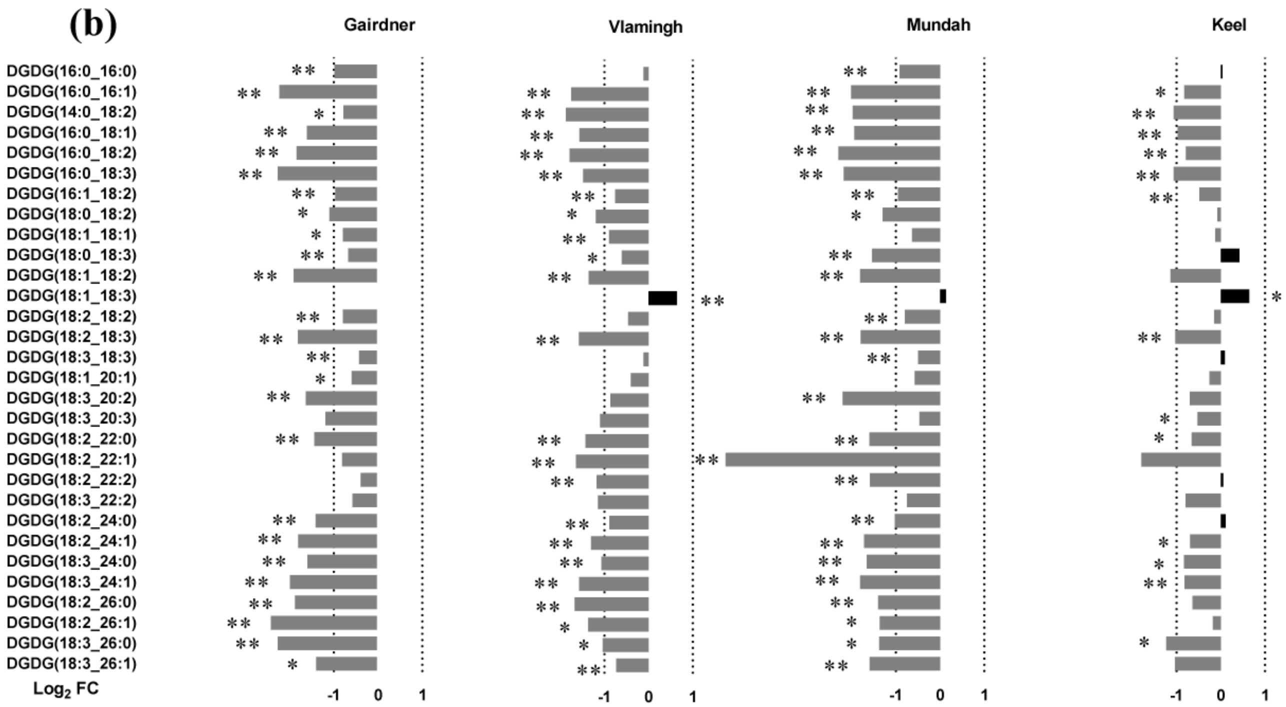
(b)
Gairdner
Vlamingh
Mundah
Keel
DGDG(16:0_16:0)
DGDG(16:0_16:1)
DGDG(14:0_18:2)
DGDG(16:0_18:1)
DGDG(16:0_18:2)
DGDG(16:0_18:3)
DGDG(16:1_18:2)
DGDG(18:0_18:2)
DGDG(18:1_18:1)
DGDG(18:0_18:3)
DGDG(18:1_18:2)
DGDG(18:1_18:3)
DGDG(18:2_18:2)
DGDG(18:2_18:3)
DGDG(18:3_18:3)
DGDG(18:1_20:1)
DGDG(18:3_20:2)
DGDG(18:3_20:3)
DGDG(18:2_22:0)
DGDG(18:2_22:1)
DGDG(18:2_22:2)
DGDG(18:3_22:2)
DGDG(18:2_24:0)
DGDG(18:2_24:1)
DGDG(18:3_24:0)
DGDG(18:3_24:1)
DGDG(18:2_26:0)
DGDG(18:2_26:1)
DGDG(18:3_26:0)
DGDG(18:3_26:1)
Log_2 FC
-1 0 1

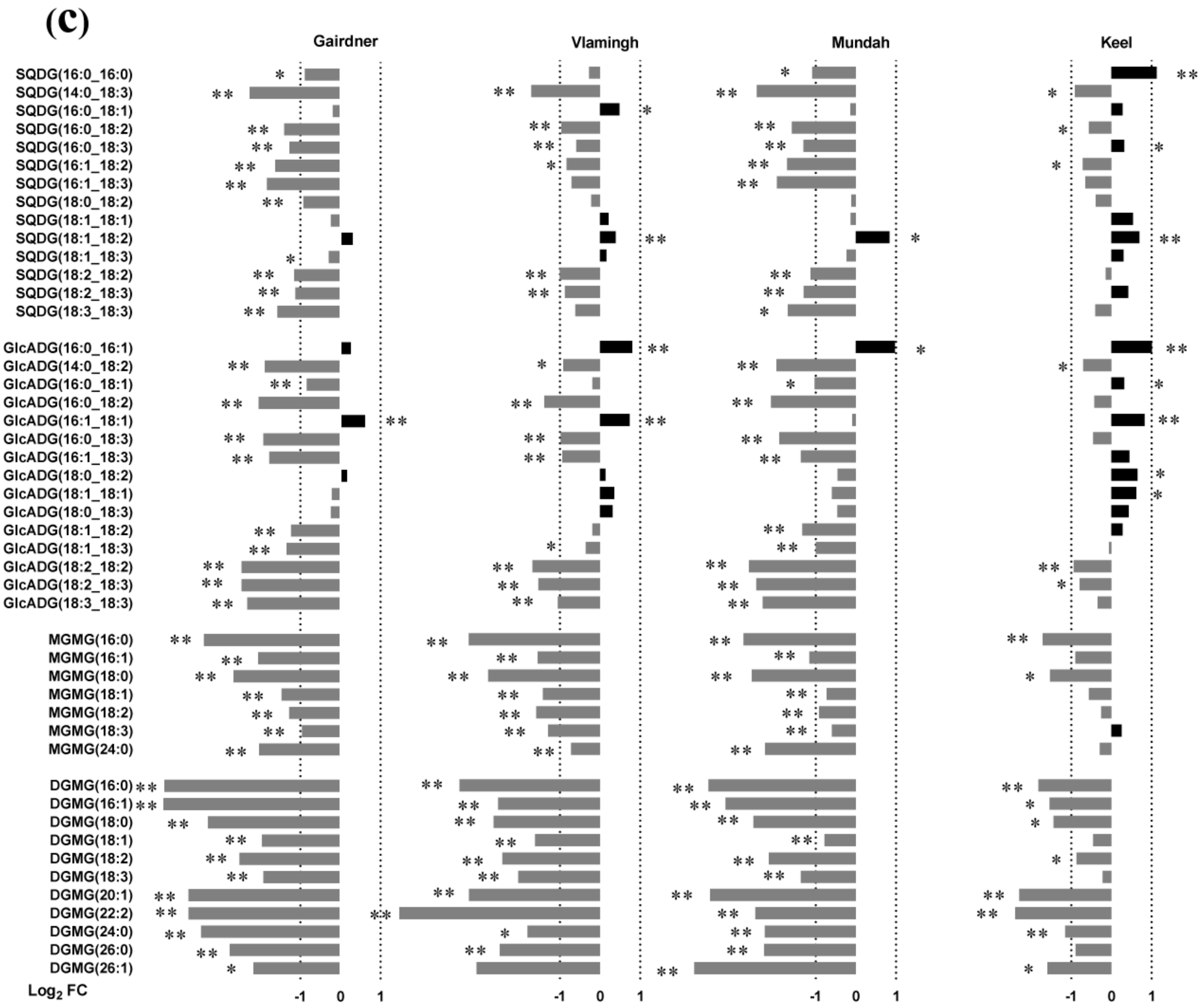

Supplementary Figure 2. Log2 fold change (FC) of plastidial glycerolipids including MGDGs (a), DGDGs (b), SQDGs, GlcADGs, MGMGs and DGMGs (c) after salt treatment in four barley varieties: Gairdner, Vlamingh, Mundah and Keel. Values with significant differ- ences between control and salt-treated samples are labelled with asterisks (*: adjusted $p < 0.05$; **: adjusted $p < 0.01$). The ±2-fold change is indicated by dashed lines.

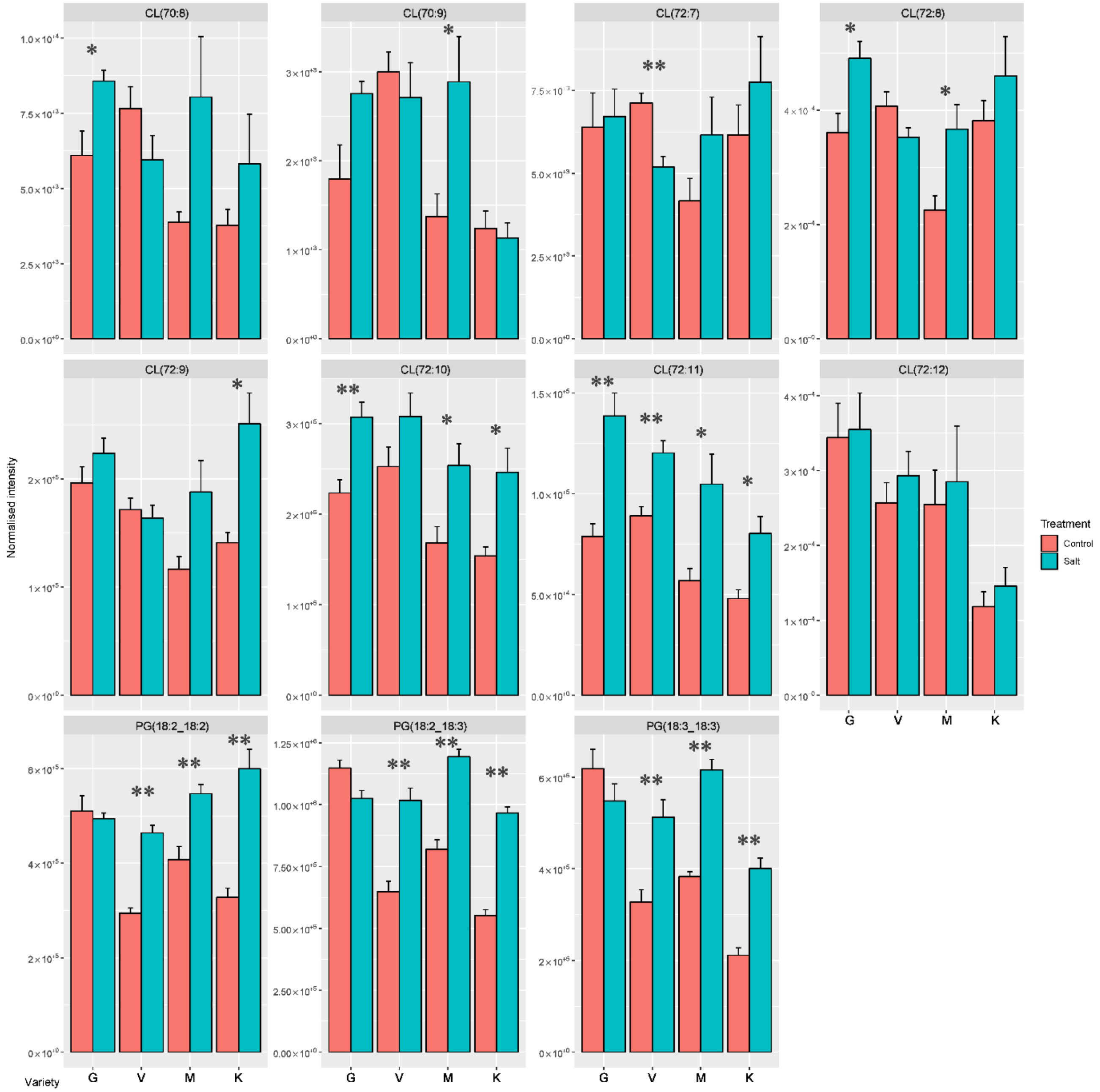

Supplementary Figure 3. Composition changes of cardiolipins (CLs) and their major biosynthetic pre- cursor — phosphatidylglycerols (PGs) after 250 mM NaCl treatment in four barley va- rieties. Gairdner (G), Keel (K), Mundah (M) and Vlamingh (V) (n = 5; values displayed as mean of normalised intensities ± SE). Values with significant differences between control and salt-treated samples are labelled with asterisks (*: adjusted $p < 0.05$; **: adjusted $p < 0.01$).
